# TMD pain: the effect on health related quality of life and the influence of pain duration

**DOI:** 10.1186/1477-7525-8-46

**Published:** 2010-05-02

**Authors:** Geerten-Has E Tjakkes, Jan-Jaap Reinders, Elisabeth M Tenvergert, Boudewijn Stegenga

**Affiliations:** 1 Department of Oral and Maxillofacial Surgery, University Medical Center Groningen University of Groningen, Groningen, PO Box 30.001, 9700 RB, the Netherlands; 2 Center for Dentistry and Oral Hygiene, Department of Oral Health Care and Clinical Epidemiology, University Medical Center Groningen, University of Groningen, Groningen, 9700 VB, the Netherlands; 3 Research Innovation Group in Health Care and Nursing, Hanze University Groningen, University of Applied Sciences, Groningen, PO Box 275, 9700 VB, the Netherlands; 4 Office for Medical Technology Assessment, University Medical Center Groningen, University of Groningen, Groningen, PO Box 30.001, 9700 RB, the Netherlands

## Abstract

**Objectives:**

As impact of literature concerning this subject is scarce, the objectives of this study were to assess whether the Health Related Quality of Life (HRQoL) is decreased in patients with painful temporomandibular disorders as compared to the HRQoL in the general population, and to evaluate to what extent pain duration affects HRQoL.

**Methods:**

Data concerning physical and mental health were retrieved from patients with painful temporomandibular disorders. Assessment tools used were: the Mandibular Function Impairment Questionnaire (MFIQ), the Short-Form-36 (SF-36), the Hospital Anxiety and Depression Schedule (HADS), and the General Health Questionnaire (GHQ). In order to examine the influence of the duration of pain on HRQoL, the total sample was divided into three different subgroups. Subgroup 1 consisted of patients with complaints existing less than one year. Patients with complaints from 1 to 3 years were allocated to the second group. The 3rd subgroup included patients with complaints longer than 3 years.

**Results:**

The total sample consisted of 95 patients (90 females and 5 males). On most physical and social functioning items, groups 2 and 3 scored significantly worse than the general population. On the other hand, none of the groups differed from the general population when comparing the mental items. Duration of pain was significantly correlated with SF-36 subscale physical functioning and the mandibular impairment.

**Conclusion:**

Patients with TMD pain less than one year score better than compared to the population norm. With a longer duration of pain, mental health scores and role limitations due to emotional problems do not appear to be seriously affected by reduced physical health, while social functioning appears to be considerably affected.

## Background

Temporomandibular disorders (TMDs) comprise a group of disorders that affect the temporomandibular joint (TMJ), the masticatory muscles, or both. TMDs involve musculoskeletal pain, disturbances in the mandibular movement patterns, and/or impairment in functional movement [1]. Pain is the main characteristic of most TMDs and also the main reason for patients to seek treatment [2]. Many TMDs should be considered chronic pain conditions, since they show lot of similarities [3]. Psychological factors have been implicated in the initiation as well in the perpetuation of several TMDs [4]. Stress, somatic distress, and depression may be potential etiological risk factors for TMD-related pain [5]. When the duration of pain increases, psychological factors may become more obvious and prominent. Even after a decrease of the somatosensory input, suffering and pain behaviour may continue and even increase [6].

It is generally accepted that quality of life is negatively affected by chronic pain [7,8]. The impact of TMDs (and other types of orofacial pain) on Health Related Quality of Life (HRQoL), however, has scarcely been described. Recently, Naito et al. conducted a systematic review on oral health status and health-related quality of life [9]. They found only one study concerning TMDs. In this study, Reisine and Weber [10] observed a sample of 30 patients with temporomandibular disorders, during 6 months. Different aspects of HRQoL were investigated e.g. anxiety, perceptions and social functioning. It was found that while the pain decreased over time, oral and functional aspects did not improve significantly within the same period of time. This result may be due to a slower response of other parameters to treatment in contrast to a relatively rapid response of pain. Furthermore, the authors found relatively poor ratings of well-being and high levels of anxiety, suggesting that TMD patients are characterized by relatively negative psychological states, and that when pain persist (even when diminished) functional aspects do not improve.

Murray et al. [11] described the HRQoL, as measured with the Oral Health Impact Profile (OHIP), of patients referred to a craniofacial pain unit because of TMD and facial pain. With regard to pain-related disability and HRQoL, 29.7% of the sample reported a frequently disturbed sleep as a consequence of their oral conditions, and 36.4% reported feelings of depression. Different researchers have found a larger impairment of the oral HRQoL in TMD patients compared with healthy population, using the OHIP [12,13].

LeResche et al. [14] studied the facial expression as well as states of anxiety, depression, somatization and daily stress in a group of TMD pain patients, subgrouped into a chronic and non-chronic category. With regard to these four aspects of HRQoL, no differences were found between a group of patients that perceived pain for the first time within the last two months (non chronic group) and a group that suffered from pain for over 6 months (chronic group).

It is not clear whether and, if so, how (chronic) pain related to TMDs influences HRQoL, and whether pain duration is of influence. It may be hypothesized that when pain has just begun, this will mainly affect physical factors such as physical functioning. When the pain lasts for a longer period, and treatment so far has failed to relieve the pain, it may start to have more impact on the emotional behaviour, social factors and HRQoL. However, whether this is the case is yet not clear. Information concerning the influence of pain and its persistence on HRQoL may guide (the emphasis of) treatment in these patients. Therefore, the aims of this study were to assess whether the HRQoL is decreased in orofacial pain patients as compared to the general population, and to study the effect of duration of pain on HRQoL.

## Methods and materials

### Sample

Patients were recruited from the department of Oral and Maxillofacial Surgery of the University Medical Center Groningen. The group consisted of 95 patients consecutively consulting the TMD/Orofacial Pain section for their orofacial pain problems. The inclusion criteria were age over 16 years, no language barrier, and the presence of a painful temporomandibular disorder as classified according to the RDC/TMD [15,16]. From the axis II information, the duration and impact of the pain were assessed. The influence of the duration of pain on HRQoL was examined by two means. Firstly, the total sample was divided into three different subgroups. Subgroup 1 consisted of patients with complaints existing less than one year. Patients with complaints from 1 to 3 years were allocated to the second group. The 3^rd ^subgroup consisted of patients with complaints longer than 3 years. Secondly, the influence of the duration of pain was studied using results of the total sample in regression analysis. During their first visit to the clinic, patients were informed about the study and the content of the questionnaires. When patients were willing to participate, they were requested to fill in an informed consent.

Quality of life has been described by the World Health Organisation as " ... an individual's perception of their position in life in the context of the culture and value system of which they live with the relation to their goals, expectations, standards and concerns." This concept incorporates different aspects of individuals, including physical health, psychological state, level of independence, social relationships, personal beliefs and their relationship to salient features of the environment [17].

### Assessment and Instruments

During the second visit to the clinic, patients were instructed how to complete the questionnaires. Subsequently, patients were left alone to complete the questionnaires. When necessary, unclear test items could be clarified by the interviewer. Only Dutch versions of the questionnaires were used.

### HRQoL was measured by the following instruments

#### SF-36

The Medical Outcome Short Form Health Survey, a 36 item health survey, was used to assess the patients' HRQoL [18]. It includes eight health concepts: *physical functioning *(PF, measuring the physical activities), role limitations due to *physical health problems *(RP, measuring the effect of the physical health on work and daily activities), *bodily pain *(BP), *general health *(GH) perceptions, *vitality *(VT, measuring energy/fatigue), *social functioning *(SF), role limitations due to *personal or emotional problems *(RE measuring the effect of the emotions on work and daily activities) and *general mental health *(MH including anxiety and depression). The scores on every subscale range from 0-100, with higher scores indicating better health states. Reference values were used to compare the results of the group in question. The reference values were taken from a Dutch study, which consisted of a random Dutch sample of 1742 persons, which is used as the reference group [19]. In this study the mean values for each subscale were for PF 83.0 (sd 22.8), RP 76.4 (sd 36.3), BP 74.9 (sd 23.4), GH 70.7 (sd 20.7) VT 68.8 (sd 19.3), SF 84.0 (sd 22.4), RE 82.3 (sd 32.9), MH 76.8 (sd 17.4) [19].

#### MFIQ

The Mandibular Function Impairment Questionnaire was used to obtain information about the function impairment of the jaw. It was developed to provide for a tool, additional to the clinical assessment, for assessment of function impairment in the patients own value system [20]. It comprises 17 items, concerning mandibular functions e.g. speaking and eating different types of food. A functional impairment rating score (FIRS) can be retrieved. This is a score ranging from 0 (no function impairment) up to 5 (indicating severe function impairment) [20].

#### HADS

To assess depression and anxiety in a hospital setting, the HADS was used [21]. To screen for anxiety (HADS-A) the odd items were used. For the screening of depression (HADS-D), the even items of questionnaire were used. On each subscale, scores up to 7 indicate no signs of anxiety or depression, scores between 8 and 10 suggest probable anxiety or depression, and scores over 10 indicate the presence of anxiety or depression, respectively [21].

#### GHQ-28

The general health questionnaire was used to assess different types of psychiatric distress. It is a 28 item list which can be divided into four different subscales: *somatic symptoms *(GHQA), *anxiety and insomnia *(GHQB), *social dysfunction *(GHQC) and *severe depression *(GHQD) [22]. The reference values were for GHQA 6.2, GHQB 5.8, GHQC 7.0, GHQD 1.6 and were retrieved from a general Dutch population of 485 persons [23].

### Sample size calculation

To estimate the a priori sample size, an effect size of 0.4 was chosen. By convention this effect size is considered as a moderate effect size. Sample was calculated on an ANOVA with the parameters α, β, number of groups and effect size. α was set at 5%, β at 10%, number of groups 3, resulting in a critical F of 3.10931. The total calculated sample was 84. To account for possible dropouts, sample size was about 10% increased to 95.

### Statistical analysis

Descriptive statistics were performed to summarize sample characteristics. Data were tested for normality using the Kolmogorov-Smirnov test. By means of one sample T-tests, the HRQoL scores of the patients were compared to those of a general population. To test mean differences in HRQoL among subgroups, one-way ANOVA was carried out, followed by Scheffe's post hoc multiple comparison test in case of a significant result. In order to study the association between the duration of pain and the scores on the different SF-36 subscales, HADS scores, GHQ-28 scores, and MFIQ score, respectively, Pearson correlation coefficients were calculated. Outlier analysis with scatter plots was performed to look for possible difference in scores between female and male participants. Data from the total sample was analysed in a regression analysis. Data were analyzed using SPSS 14 (SPSS Inc, USA). The level of significance was set at 0.05. This study was approved by the Medical Ethical Committee of the University Medical Center Groningen.

## Results

### Patients

In total 95 patients (90 females and 5 males) provided their consent to participate in the study. Their average age was 40.3 yrs (sd 13.1, ranging from 17-69). According to the RDC/TMD criteria, patients were diagnosed with a group I diagnosis (myofascial pain), a group II diagnosis (disc displacement) a group III (arthralgia, osteoarthritis and osteoarthrosis). A group I diagnosis was established in 31.9%; a group II diagnosis in 4.4% and a group III in 35.2%. A combined diagnosis was made in 28.7% of all cases (in 7.8% group I and II, in 17.6% group I and III, and in 3.3% group II and III were combined). Furthermore, the participants of the 3 subgroups based on pain duration were calculated: subgroup 1 (pain present for less than one year) consisted of 15 patients (14 females; 1 male, mean age 37.7 yrs, sd 14.4, range 17-69), subgroup 2 (1-3 years pain duration) consisted of 16 patients (13 females, 3 males; mean age 37.5 yrs, sd 14.1, range 20-68), and subgroup 3 (more than 3 years of pain) consisted of 64 patients (63 females, 1 male; mean age 41.6 yrs, sd 12.5, range 17-67). The distribution of the diagnoses, medication use and coinciding chronic pain diseases among the three groups is listed in Table 1.

**Table 1:** Distribution of age, RDC diagnoses medication usage and coinciding chronic pain disorders among the three subgroups.

	Group		
	1	2	3
Total	15	16	64
Female/male	1/14	3/13	1/63
Age (sd)	37.6 (14.4)	37.5 (14.1)	41.6 (12.5)
*RDC/TMD diagnosis:*	n	n	n
Group I	3	8	20
Group II	0	0	3
Group III	5	7	24
Group I + group II	0	0	4
Group I + group III	5	0	12
Group II + group III	2	1	1
			
*Analgesic usage*			
Paracetamol	0	1	7
NSAID	1	0	3
Tricyclic antidepressant	1	5	6
Tricyclic antidepressant + paracetamol	1	0	0
Tricyclic antidepressant + NSAID	0	0	2
NSAID + opioid	0	0	2
*Other chronic pain condition*			
Rheumatoid arthritis	0	2	1
Hernia	0	0	1
Back pain	0	0	1

Group 1: Myofascial pain

Group 2: Disc displacement

Group 3: Arthralgia/osteoarthritis/osteoarthrosis

NSAID: Non-steroidal anti-inflammatory drug

### Effects of pain duration: Three groups

#### Results compared with reference values

Table 2 shows the mean SF-36 and GHQ scores for the three subgroups. The first ("relatively acute") subgroup scored better on the subscale *physical functioning *and worse on subscales *general health *and *vitality *than the general population, but on the other subscales this subgroup and the general population revealed comparable scores.

**Table 2:** SF-36 and GHQ scores in three groups of orofacial pain patients and reference values.

	reference	1	2	3
Scale	Mean (SD)	Mean (SD) n = 15	Mean (SD) n = 16	Mean (SD) n = 64
SF-36 PF^a^	83.0 (22.8)	92.1 (8.1)*^3^	74.2 (33.1)	72.3 (25.7)*^1^
SF-36 RP^a^	76.4 (36.3)	54.2 (38.1)	71.2 (3.6) ^3^	41.2 (39.7)*^2^
SF-36 BP^a^	74.9 (23.4)	66.1 (20.4)^3^	53.2 (27.5)*	48.5 (19.7)*^1^
SF-36 GH^a^	70.7 (20.7)	53.0 (23.4)*	55.8 (18.7)*	54.4 (20.6)*
SF-36 VT^a^	68.8 (19.3)	52.9 (14.9)*	49.3 (21.6)*	55.2 (19.4)*
SF-36 SF^a^	84.0 (22.4)	74.0 (22.9)	66.0 (29.2)*	66.7 (23.4)*
SF-36 RE^a^	82.3 (32.9)	66.7 (31.8)	81.0 (55.0)	80.9 (34.1)
SF-36 MH^a^	76.8 (17.4)	66.4 (12.5)	60.6 (17.9)^3^	73.2 (13.0)^2^
GHQA^b^	6.2	6.7 (4.1)	6.3 (4.1)	8.2 (4.0)*
GHQB^b^	5.8	2.8 (2.1)^2^	7.5 (3.6)^1^	5.9 (4.4)
GHQC^b^	7.0	6.6 (1.8) ^3^	8.2 (3.3)	8.3 (2.3)*^1^
GHQD^b^	1.6	0.8 (1.8)	2.7 (3.5)	1.9 (3.0)

Values in mean (standard deviation, * p < 0.05 from reference values, in superscript the group to which the value is statistically different; reference values from ^a ^Aaronson et al.[19], ^b ^Koeter & Ormel [23].

(PF, physical functioning; RP, Role limitations physical; BP, bodily pain; GH, general health; VT, vitality; SF, social functioning; RE, role limitations emotional; MH, mental health; SD, standard deviation; GHQA, somatic symptoms; GHQB, anxiety and insomnia; GHQC, social dysfunction; GHQD, severe depression; P, P-value)

1. group with complaints of one year or shorter

2. group with complaints within 1 and 3 years

3. group with complaints longer than 3 years.

Compared with the general population, the second subgroup scored worse on four subscales (*bodily pain, vitality, general health *and *social functioning*) and the third subgroup scored worse on six SF-36 subscales (*bodily pain, vitality, general health, social functioning*, *physical functioning *and *role emotional*).

In the first and second group, scores on the GHQ scales did not significantly differ from scores in the general population. The third group showed more impairment in somatic symptoms and showed higher social dysfunction, with worse scores on GHQA and the GHQC, compared to those obtained from the general population.

#### Comparisons between groups

No differences were found in age between the three groups. Statistically significant differences were found between groups 1 and 3 with regard to the SF-36 subscore on the scales *physical functioning *and *bodily pain *(i.e. scores were better in group 1), but not between groups 2 and 3. Other SF-36 scores did not differ significantly between the three groups.

The third group showed more somatic problems as well as a higher level of social dysfunction compared to the first group, as GHQA and GHQC scales revealed significant differences between these groups.

The patients' impairment in mandibular function, as assessed with the MFIQ and expressed in the function impairment rating scale (FIRS), was 2.4 (sd 1.1) for the first group, 2.6 (sd 2.0) for the second group, and 3.3 (sd 1.6) for the third group, indicating moderate impairment in these three subgroups (Table 2). No significant differences between the three groups were found in the FIRS.

Both HADSA and HADSD scores were worse in groups 2 and 3 as compared to group 1 (Table 3). In addition, the HADSD score in group 2 was worse than in group 3.

**Table 3:** FIRS, HADSA and HADSD scores (standard deviation) for three groups.

	Group		
	1	2	3
score			
FIRS (sd)	2.4 (1.1)	2.6 (2.0)	3.3 (1.6)
HADS A (sd)	3.3 (1.8)^2,3^	6.4 (4.1)^1^	5.0 (3.6)^1^
HADS D (sd)	1.6 (1.1)^2,3^	6.2 (4.2)^1,3^	3.5 (3.1)^1,2^

1.: group with complaints of one year or shorter

2.: group with complaints within 1 and 3 years

3.: group with complaints longer than 3 years.

(FIRS; functional impairment rating score, HADSA; anxiety, HADSD; depression. In superscript the group to which the value is statistically different)

### Effects of pain duration: total sample

The social, psychological and part of the physical measures did not show significant correlation with pain duration. Of all calculated correlations, the SF-36 subscale *bodily pain *and the Function Impairment Rating Scale (FIRS) were significantly correlated with the duration of pain.

Outlier analysis revealed no differences on the subscales in any of the assessed subscales between female and male patients.

## Discussion

In this study we examined whether the duration of pain in TMD patients seeking treatment affects the HRQoL and psychological well-being. When managing these patients, psychological assessment may lead the clinician to multidimensional, biobehavioral therapy modalities rather than to somatically based therapies [2]. Also, TMD patients classified into different cognitive-behavioural profiles seem to respond differently when the same treatment is offered [24]. Thus, not only the physical but also the psychological status may influence the treatment outcome.

**Figure 1:**
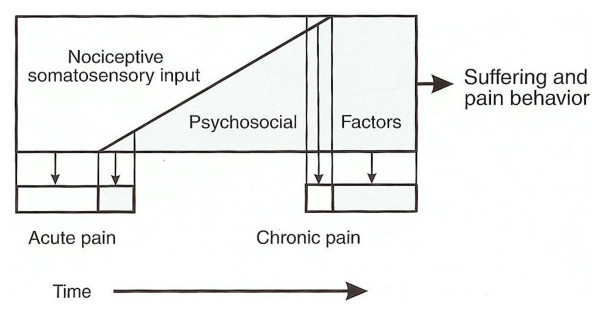
**The effect of duration of symptoms on psychosocial factors**. From Okeson [6]. Used with permission.

The duration of pain is thought to have a significant impact on a patient's psychological status (Figure 1) [6]. To provide more insight into the effect of duration of pain complaints, we compared patients with relatively acute pain (less than 1 year) and patients with chronic pain (1-3 years and > 3 years, respectively). A striking finding was that in all three subgroups the SF-36 scores of the scales *role emotional *and *mental health *did not differ significantly from the reference values. It may be, that patients with a longer experience of complaints tend to get used to their pain and symptoms and incorporate them as a part of their life, thereby leaving their mental health less affected.

The first group did score significantly higher ("better") on the scale "physical functioning", compared to the population norm. This finding could be explained by possible underestimation of the physical situation, when patients visit a hospital and are allocated to a study group that answers questions about health. Patients with pain or function problems that have arisen within the last year, may tend to focus on these problems in an opposite manner than patients who have a longer experience with these problems. In addition, the scale bodily pain was not significantly different from the reference value. Because of the relatively short existence of pain, patients may underestimate or underrate the consequences of their disorders on the measured scales. They may be convinced that (pain) symptoms will be transient and, therefore, patients will not allow them to affect the physical items. Patients may also feel the need to convince the doctor that the symptoms are purely physical, and want to display that statement in the answers of the subscales.

By contrast, on SF-36 physical health items ("*bodily pain*", "*general health*" and "*vitality*") the second and third group scored significantly worse than reference values. In addition, in the third group the items *physical functioning *and *role physical *were also worse than reference values. This physical impairment was confirmed by the FIRS scores, indicating moderate function impairment. So the mandibular function was lowered in all three groups. The GHQA score, which represents the somatic general health, is significantly lower ("worse") in the third group, which is in accordance with the scores on the SF-36. With significantly lower scores on the physical scales of the SF-36, the second and third subgroup did not score significantly lower on the mental scales.

Between the different pain duration groups, statistically significant differences were found only on a few scales. Comparing "better" scores from the first group with "worse" scores from the third group, leads in the *physical functioning *scale to a significant difference between those scores. Although the HADS scores are interpreted using cut-off points, it is striking that the depression scale (HADSD) in group 2 is not only higher compared to group 1, but also compared to group 3. Patients who experience complaints for a short time may not be seriously affected, but when pain persists, psychological distress will be more pronounced. Later, when patients are used to the pain or when they are sufficiently reassured about their health status, the psychological distress will return to lower values again. This is in accordance with the score on the mental health item of the SF-36, which is better in the third group compared to the second group.

According to our findings, it may seem that patients with a shorter duration of pain seem to underrate their physical impairment or at least do not consider it to be relevantly impaired, as the scores are "better" compared with a healthy reference group. Patients with longer lasting pain at least longer than one year, have more pronounced physical problems. The role limitations due to emotional problems or the mental health seem to be hardly affected, however. It has been suggested that psychological functioning is merely related to patients' beliefs and coping strategies rather than to the physical impairment [25]. On the other hand, the social functioning scale in the SF-36 as well as the GHQC score suggest that social functioning is affected in the third group. This may be explained by role limitations due to physical limitations, which in turn may be the result of the actual disorder.

In addition to the analysis with three subgroups, we calculated Pearson correlation coefficients with data from all patients. This revealed a significant correlation between the whole range duration of pain with the subscale *physical functioning *and the mandibular impairment (FIRS). So with a longer duration of pain, the somatic well-being is considered worse. It remains unclear whether the physical discomfort has worsened during its existence or whether the discomfort is only rated worse due to its longer existence. Other subscales and other scores did not show significant correlation with duration of pain, which may be explained by a large range of duration of pain in contrast to the smaller scale range of the scores on other subscales and the other questionnaires.

One factor that may be of influence on the results is the age. In our sample, no difference was found between the three groups. Besides TMD pain, other pain condition could play a role in HRQoL. Of the studied sample totally five had an accompanying chronic condition. In the second group, two suffered also from rheumatoid arthritis. In the third group, one patient suffered from rheumatoid arthritis, one from hernia and one from low back pain. These conditions could have influenced the questionnaire outcomes, although the number of patients is a slight minority compared to the total sample size, therefore we argued this to be of negligible influence.

A limitation of this study could be the large female predominance, which is than in the general population, which may hamper the generalizability of the results. However, a predominance of female gender in TMDs is also found in epidemiological research [26]. In addition, more female than male patients seek treatment for their pain problems, leading to an increasing female predominance in specialist centers, with a female:male ratio ranging from 2:1 to 9:1 [27]. In addition, outlier analysis (to explore possible differences in measurements in our sample between male and female patients) revealed no outliers in the assessed subscales. We thus consider sex difference in our sample to be of minor influence and we therefore decided to include both male and female in the total analysis.

## Conclusion

In patients with chronic pain conditions, such as most TMD pains, it has been demonstrated that psychological factors are better predictors of treatment outcome on the long-term than physical findings are [2,28]. When TMD patients with pain less than one year are compared to a reference population, it was found that these patients scored better on physical functioning. However, we found that patients with longer lasting problems have more pronounced physical problems and limitations and that these limitations have impact on social functioning in this group. The mental health and role limitations due to emotional problems do not seem to be seriously affected by reduced physical activities. Especially in cases of longer duration of pain, where initial treatment has failed to relieve the pain, the social functioning may be considerably affected and should therefore be taken into account when managing these conditions.

## Competing interests

The authors declare that they have no competing interests.

## Authors' contributions

GHET conceptualized and designed the study, acquired the data and participated in data analysis, and drafted the manuscript;

JJR, ETV, BS contributed in data analysis and participated in revising the manuscript critically for important intellectual content.

All authors read and approved the final manuscript.
